# Physical fitness as a predictor of health-related quality of life in children

**DOI:** 10.3389/fspor.2026.1860770

**Published:** 2026-06-18

**Authors:** Marko Badrić, Igor Vrga, Leona Roca Badrić

**Affiliations:** 1 Faculty of Teacher Education, University of Zagreb, Zagreb, Croatia; 2 General Hospital Sisak, Sisak, Croatia

**Keywords:** cardiorespiratory fitness, children, health, muscular fitness, physical fitness, quality of life

## Abstract

The aim of this study was to examine the association between the components of physical fitness and health-related quality of life (HRQoL) in young school-age children and to determine the contribution of physical fitness in predicting children's subjective well-being and quality of life. The study initially included 306 primary school students (163 girls and 143 boys), with an average age of 9.89 ± 0.60 years. Body height was measured with a portable altimeter, and body mass, body mass index (BMI) and body fat percentage were measured with dual-frequency body composition analyzer. Physical fitness was measured with the hand taping, long jump, trunk lift, 10 × 5 m run and Shuttle run tests, while quality of life was assessed with the KIDSCREEN-10 questionnaire. To identify predictors of quality of life, hierarchical regression analysis was performed; In the first step, control variables (gender, age, and BMI) were included in the model, while in the second step, physical fitness variables were added. Significant gender differences in speed and explosive power were found in favor of boys. Hierarchical regression analysis showed that demographic and morphological variables (age, gender, BMI) were not significant predictors of quality of life. However, by adding physical fitness components, the model becomes statistically significant (*R* = 0.266; *R*^2^ = 0.071; *p* = 0.005), with a total of 7.1% of the variance in quality of life explained. The change in variance between the first and second models is Δ*R*^2^ = 0.068 (*p* = 0.001). Cardiorespiratory endurance (VO_2_max) and speed of simple movements were identified as the most significant predictors. While simple movement speed was positively correlated with higher quality of life, cardiorespiratory endurance emerged as a negative predictor in this specific sample. The results obtained confirm that physical fitness contributes to predicting children's subjective well-being, and its components are more important indicators of health and quality of life although they explain a modest proportion of the overall variance. These findings suggest that school-based Physical Education programs should continue to support the systematic and balanced development of aerobic and fundamental motor skills.

## 1 Introduction

Being physically fit in childhood is increasingly recognized as an indicator of current and future health status and is no longer seen solely as a result of playing sports (1). The main components of physical fitness are cardiorespiratory fitness, muscle strength, and motor fitness (1, 2). Physical fitness is considered an important health marker, both in early years and later in life, and has a direct association with better health outcomes (1, 3, 4). The period between the ages of nine and eleven is an important developmental period because it is when levels of physical activity and movement patterns are formed that can have long-term developmental consequences in the child's life, from anthropometric, cardiometabolic to mental (3–5). Physical fitness associated with health is the ability to perform physical activity that integrates most of the body's functions involved in body movement (6).

The World Health Organization has defined health-related quality of life as a multidimensional and integrative construct consisting of physical, psychological and social well-being and functioning (7). Quality of life has a physical component that includes aspects such as health, nutrition, as well as protection from pain and disease (8). Moderate and high levels of physical activity (PA) are associated with higher quality of life, and physical activity may be particularly effective in improving positive well-being through health promotion, not just symptom reduction (9, 10). Systematic assessment of health-related quality of life in the general population of children and adolescents is extremely important in order to identify children at risk of low quality of life as early as possible (11).

Physical fitness is associated with health-related quality of life, especially among young people (12). Physical fitness affects an important component of quality of life related to mental health. It increases self-esteem, self-confidence and reduces symptoms of depression, which is increasingly present among children and adolescents (13). Research shows that cardiorespiratory endurance and muscle strength, or the overall status of physical fitness, are associated with the quality of life of children and young people (14). A systematic review shows that there is consistent evidence to support the claim that healthy children and adolescents with high levels of cardiorespiratory and muscular fitness have better health-related quality of life (HRQoL) than their peers who do not, demonstrating greater physical and psychological well-being and better peer relationships (15). Higher levels of cardiorespiratory endurance more consistently predict better quality of life compared to other components of physical fitness such as muscle strength and speed or agility (16–18). In order to achieve a good quality of life in children aged 9–11, basic motor skills and motor abilities themselves play an important role (3, 19). Fundamental motor skills and advanced motor abilities may be directly associated with higher quality of life, while physical fitness—particularly cardiorespiratory fitness—may act as a mediator in the mutual proportions of these two relationships (20, 21).

The aim of this study was to determine the association between the components of physical fitness and quality of life in children and to determine which components of physical fitness significantly predict quality of life after controlling for gender, age and body mass index (BMI). The specific objectives of the study relate to examining gender differences in physical fitness and quality of life and determining the association between individual components of physical fitness (cardiorespiratory endurance, explosive strength, repetitive strength, agility and speed) and quality of life.

## 2 Materials and methods

### 2.1 Sample of respondents

This cross-sectional study involved 306 elementary school students from the continental part of the Republic of Croatia, of whom 163 were girls and 143 were boys. The average age of the respondents was 9.89 ± 0.60. The criteria for inclusion in the study were students who met the following conditions: completely healthy without physical and mental disabilities and regular attendance of physical education classes, signed parental consent, and completed measurements in all morphological and motor tests, as well as questionnaires that were used for further analysis. The procedures carried out in the study were anonymous and were conducted in accordance with the Declaration of Helsinki. The study was approved by the Ethics Committee of the Faculty of Teacher Education, University of Zagreb (protocol code 641-03/24-01/02; Reg. No. 251-378-01-24-2).

### 2.2 Morphological measurements

Body height was measured by a standardized anthropometric procedure using a high-precision portable height gauge (Seca® 213, Hamburg, Germany) with an accuracy of 0.1 cm, which is in accordance with common protocols for assessing growth in young school-age children (22, 23). Body mass and body composition (total body mass, body fat percentage, lean mass) were assessed using a dual-frequency TANITA DC-360P body composition analyzer (Tanita Corporation, Japan), which is based on the principle of bioelectrical impedance (BIA). BIA is a commonly used, practical, and child-friendly method for assessing body composition in school-age children (24–27). Validation studies in children aged approximately 6–12 years show very high reliability and strong correlation with reference methods (DXA, isotopic dilution), with a tendency to underestimate fat mass and body fat percentage, so the results are recommended to be interpreted with caution (28–30). Body mass index (BMI) is calculated as the ratio of body mass (kg) to the square of body height (m^2^). Despite the limitation of not distinguishing fat and lean mass, BMI remains the most commonly used indicator of nutritional status and overweight in younger school-age children in large epidemiological and school-based studies (31–35).

### 2.3 Measurement of cardiorespiratory fitness

Cardiorespiratory fitness was assessed by a multi-stage 20-meter shuttle run test. In this field test, subjects run back and forth between two set lines at a distance of 20 m. Running speed is regulated by audible signals and starts at 8.5 km/h^−1^ and increases by 0.5 km/h^−1^ every minute. Each level lasts approximately 60 s, with the “speed” (the duration of each interval) dictated to the subject by the interval of audible signals (36). The task of the measurement is for the subject to maintain the given audible running rhythm for as long as possible. Maximal oxygen consumption (VO_2_max, mL·kg⁻¹·min⁻¹) was calculated using the equation: VO_2_max = 31.025 + 3.238S−3.248A + 0.1536(A × S), where S is the speed in kilometers per hour achieved at the end of the test, and A is the age in years (37). The equation is suitable for boys and girls aged 8–19 years. The measurement is interrupted when the subject fails to maintain the given audible rhythm twice in a row or gives up on his own due to the inability to continue running. Testing of students was carried out in school sports halls.

### 2.4 Motor skills measurement

Motor skills in early school age are often measured with short tests that include speed, strength, agility and coordination. Numerous studies in primary school children in Europe use the standing long jump and shuttle run tests (4 × 10 m or 10 × 5 m) as standard indicators of motor readiness. Motor skills in early school-age children assessed with these four motor tests have been widely applied in the primary school population (38–41). The speed of simple hand movements was measured with the Hand Tapping Test, which assesses the speed of repeated movements of the upper limb and is often included in motor tests for school children. The explosive power of the lower extremities was tested with the Standing Long Jump Test, a standard field test for assessing leg muscle strength in children and which is regularly used in school and national research. Repetitive trunk strength was assessed with the Sit-ups test, which is an integral part of several standardized batteries for assessing students' motor skills and physical fitness. Agility and speed of movement were measured with the 10 × 5 m Run test, a form of the shuttle-run test that requires rapid changes of direction and combines speed and coordination; similar protocols (4 × 10 m, 10 × 5 m) are recommended and used to assess motor readiness in primary school age.

### 2.5 Quality of life

The quality of life of the respondents was assessed using a questionnaire for children and adolescents aged 8–18 years The KIDSCREEN Group Europe (7). To assess the subjective health and well-being of adolescents, the Croatian version of the KIDSCREEN-10 quality of life questionnaire was used, which is a short form of the KIDSCREEN-52 questionnaire. The Croatian version of the questionnaire shows satisfactory metric characteristics and allows researchers to reliably assess quality of life in all age groups (42). The questionnaire assesses the dimensions of physical and psychological well-being, autonomy and parental relationship, peer and social support, and school environment. The questionnaire consists of 10 questions in which respondents indicate their degree of agreement with the content of each statement on a five-point Likert-type scale, and in the end, one global result is obtained. The metric characteristics of the KIDSCREEN-10 questionnaire are at a satisfactory level. Cronbach's alpha values are 0.82, and the test-retest coefficient is 0.70, which is a satisfactory result of the internal consistency of the questionnaire (43).

### 2.6 Statistical analyses

The basic statistical parameters arithmetic mean, standard deviation, skewness and kurtosis were calculated. Differences in anthropometric characteristics, physical fitness components and quality of life by gender were analyzed using the *t*-test for independent samples, with a previous check for homogeneity of variance using Levene's test. Pearson's correlation coefficient was used to examine the relationship between quality of life and physical fitness components.

To determine the predictors of quality of life, hierarchical regression analysis was performed. The statistical significance of the regression models was assessed using the *F*-test (ANOVA of the regression model). In the first step, control variables (gender, age and body mass index) were included in the model, while in the second step, physical fitness variables (cardiorepiratory endurance, agility, explosive strength, repetitive strength and speed) were added. Before conducting the statistical analysis, the assumptions of the regression analysis were checked. The results of the Shapiro–Wilk test indicated deviations from normal distribution for most variables (*p* < 0.05). The normality of the distribution of residuals was checked by visual inspection of the histogram and Q-Q plots. The results showed that the residuals followed an approximately normal distribution without significant deviations from the diagonal line. Multicollinearity among predictors was checked using the variance inflation factor (VIF). The values obtained ranged between 1.031 and 1.224, indicating the absence of multicollinearity problems (44). The independence of the residuals was assessed using the Durbin–Watson test, with a value of 1.83 obtained, indicating the absence of autocorrelation and satisfaction of the assumption of independence of errors (45). Given the sample size and the observation of the histogram and Q-Q plots that did not show significant deviations from normality, as well as the robustness of the parametric tests, the assumption of normality is considered acceptable (45).

Statistical data processing was performed using the IBM SPSS Statistics software package, version 29.0. The statistical significance level was set at *p* < 0.05.

## 3 Results

The results in Table 1 show the basic descriptive indicators of the analyzed variables. The respondents were on average 145.31 cm tall and weighed 40.56 kg. The average body mass index (BMI) was 18.83 ± 4.20, and the proportion of body fat was 20.72%. The results of the physical fitness tests indicate an average distance traveled of 185.69 m (SD = 83.53), and a long jump of 137.92 cm. The overall assessment of the quality of life was relatively high (M = 4.21 ± 0.53). The values of asymmetry and flattening indicate an approximately normal distribution of most variables, with slightly positive asymmetry in body mass and body mass index and negative asymmetry in quality of life.

**Table 1:** Descriptive statistics of anthropometric characteristics, physical fitness variables, and quality of life.

Variable	Mean	SD	Skew	Kurt
Body height (cm)	145.31	8.23	−0.08	−0.17
Body mass (kg)	40.56	11.96	1.09	0.90
Body fat percentage (%)	20.72	8.85	0.59	−0.28
Body mass index	18.83	4.20	1.11	0.93
Running distance-(m)	185.69	83.53	0.68	0.78
Hand tapping (f)	22.02	3.86	0.38	−0.35
Standing long jump (cm)	137.92	21.59	−0.35	1.85
Sit ups (number)	32.33	8.19	0.15	−0.14
10 × 5 m Shuttle Test (sec)	26.31	3.85	0.14	0.90
Total assessment of quality of life	4.21	0.53	−0.95	1.05

MEAN, arithmetic mean; SD,  Standard deviation; Skew, Skewness; Kurt, Kurtosis.

Table 2 shows the results of the *t*-test for independent samples, which showed the existence of statistically significant gender differences in individual components of physical fitness. Significant differences were found in the taping arm test and long jump, where boys were more dominant. Also, in morphological measures, significant differences were found in body mass index (BMI) and body weight, where it was found that girls had significantly lower values than boys. On the other hand, no statistically significant differences were found in cardiorespiratory endurance and trunk lifting. Likewise, it was found that there was no difference in quality of life between the sexes, which justifies the inclusion of gender as a control variable in further analyses.

**Table 2:** Sex differences in anthropometric characteristics, physical fitness, and quality of life (independent samples *t*-test).

Variable	*N* = 163 Girl		*N* = 143 Boys			
	Mean	SD	Mean	SD	Leven-p	*t*-test
Body height (cm)	144.24	8.14	146.52*	8.19	0.59	0.015
Body mass (kg)	38.86*	10.76	42.51	12.95	0.03	0.008
Body fat percentage (%)	21.57	8.83	19.76	8.81	0.84	0.073
Body mass index	18.31*	3.89	19.42	4.47	0.11	0.020
Running distance-(m)	179.39	78.61	192.87	88.54	0.18	0.159
Hand tapping (f)	21.63	3.90	22.48*	3.77	0.68	0.054
Standing long jump (cm)	135.33	21.70	140.88*	21.15	0.81	0.025
Sit ups (number)	32.00	8.25	32.71	8.13	0.78	0.448
10 × 5 m Shuttle Test (sec)	26.67	3.96	25.90	3.70	0.60	0.083
Total assessment of quality of life	4.20	0.55	4.22	0.51	0.36	0.712

MEAN, arithmetic mean; SD, Standard deviation; Levene-p, significance of Levene's test for equality of variances; *t*-test = *p < 0.05.

Pearson's correlation analysis (Table 3) revealed a significant but weak negative association between cardiorespiratory endurance and quality of life (*r* = −0.158). Weak positive correlations were found with speed (*r* = 0.177) and repetitive strength (*r* = 0.113). No significant correlations were found for explosive strength and agility.

**Table 3:** Pearson correlations between quality of life and physical fitness variables.

Variable	Running distance	Hand tapping	Standing long jump	Sit ups	10 × 5 m Shuttle Test
Quality of life	-,158^b^	,177^a^	,089	,113^b^	-,067

a Correlation is significant at the 0.01 level.

b Correlation is significant at the 0.05 level.

A hierarchical multiple regression analysis was conducted to examine the contribution of the physical fitness components in explaining quality of life. In the first model (Table 4), which included the control variables gender, age and BMI, no statistically significant association with quality of life was found (*p* = 0.811). This model explained only 0.3% of the variance in quality of life, indicating a very weak contribution of the control variables. In the second model, the physical fitness components consisting of cardiorespiratory endurance, speed, explosive power, repetitive strength and agility were included, and the model became statistically significant (*p* = 0.005). In total, 7.1% of the variance in quality of life was explained in this model. The change in variance between the first and second models was Δ*R*^2^ = 0.068, and statistical significance was found.

**Table 4:** Summary of hierarchical regression analysis predicting quality of life.

Model	R	R^2^	Adjusted R²	SE	*Δ*R²	Sig. *Δ*R²
1	0.056	0.003	−0.007	0.570	0.003	0.811
2	0.266	0.071	0.046	0.555	0.068	0.001

*R,* coefficient multiple correlation; R^2^, Coefficient of determination; Adjusted R^2^, Adjusted R Square; SE, Std. Error of the Estimate *Δ*R^2^, R Square Change; Sig. *Δ*R^2^, Sig. F Change.

The results of the ANOVA analysis showed that the first model was not statistically significant (*F*(3,302) = 0.32; *p* > 0.05), while the second model was statistically significant (*F*(8,297) = 2.833; *p* < 0.01). The results of the hierarchical multiple regression analysis in Table 5 showed that the control variables (gender, age and BMI) do not significantly contribute to the explanation of quality of life. By adding physical fitness variables to the model, it becomes statistically significant (*F*(8,297) = 2.833) and explains 7.1% of the variance in quality of life. Cardiorespiratory endurance (*β* = −0.190) and simple movement speed (*β* = 0.148) were shown to be significant predictors of quality of life. Other variables that make up the physical fitness component and control variables (gender, age, and BMI) were not statistically significant. Cardiorespiratory endurance had the largest relative contribution to explaining the variance in quality of life.

**Table 5:** Regression coefficients for predictors of quality of life.

	Unstandardized		Standardized			*95% CI for B*	
	B	SE	*Β*	t	*P*	*LB UB*	VIF
Gender	0.015	0.066	0.013	0.221	0.825	−0.115, 0.144	1.067
Age	−0.070	0.054	−0.074	−1.295	0.196	−0.177, 0.036	1.031
BMI	−0.002	0.008	−0.016	−0.267	0.789	−0.018, 0.014	1.105
Distance	−0.001	0.000	−0.190	−3.264	0.001*	−0.002, −0.001	1.078
Hand tapping	0.022	0.009	0.148	2.525	0.012*	0.005, 0.039	1.095
Standing long jump	0.001	0.002	0.047	0.761	0.447	−0.002, 0.004	1.224
Sit ups	0.002	0.004	0.033	0.554	0.580	−0.006, 0.011	1.158
10 × 5 m Shuttle Test	−0.010	0.009	−0.068	−1.146	0.253	−0.027, 0.007	1.113

*β*, standardized regression coefficient; SE,  standard error for beta coefficient; B , unstandardized regression coefficient; t-value; p, value for significance of whole model;VIF, Variance Inflation Factor; CI, Confidence Interval, *p < 0.05. LB, lower bound; UB, upper bound;.

## 4 Discussion

The aim of this study was to examine the association between physical fitness and quality of life in children aged 9–11 years. The results showed that physical fitness components are significant predictors of the quality of life and well-being of children, although the direction of these associations varies among specific fitness domains. The results of this study showed relatively weak but statistically significant associations between individual components of physical fitness and quality of life. New research shows that the association between physical fitness and quality of life is stable but also relatively weak, which is explained by the fact that quality of life is a complex and multidimensional construct that encompasses a wide range of physical, psychological and social factors (15, 46, 47). Also, recent research indicates that physical activity and physical fitness explain a relatively small part of the variance in quality of life (about 5%–10%), which is in line with the results obtained in this study (48, 49).

The results showed partial gender differences in the components of physical fitness, which is in line with recent research confirming the existence of gender differences in motor abilities in children. Boys achieved better results in tests of simple movement speed (hand tapping) and explosive strength (standing long jump), which can be linked to the findings that boys at that age generally achieve higher values in tests of explosive strength, speed and power compared to girls (50). The study found no significant gender differences in cardiorespiratory endurance (20-m shuttle run) and repetitive trunk strength (sit-ups), suggesting that these abilities are equally developed in both sexes between the ages of nine and eleven. In contrast, previous studies have consistently shown that boys perform better in tests of strength, speed, and cardiorespiratory endurance, while differences in some other abilities are less pronounced (13, 51). Despite differences in motor performance on simple movement speed and explosive strength tests in favor of boys, both sexes assessed their quality of life equally, which is consistent with findings that clearer gender differences in quality of life only emerge in older school age and adolescence, where girls more often report lower physical and psychological well-being and lower overall quality of life scores compared to boys (52–55). At the age of 9–11, subjective well-being and quality of life are generally high, and gender differences are only beginning to be apparent, with quality of life gradually decreasing with increasing age, especially in girls (53–55).

Likewise, a positive association between quality of life and speed and repetitive strength tests was found, which is consistent with the findings of previous research that muscle strength and speed are associated with better physical, psychological, and social aspects of quality of life in school-age children (6, 15). These results suggest that motor efficiency and a sense of physical strength can directly contribute to a child's self-confidence and more successful functioning in the school environment, which is supported by findings of a positive association between muscular and cardiorespiratory fitness with psychological well-being and better school functioning (21, 56). The interesting finding of a negative association between cardiorespiratory capacity and quality of life deviates from most recent research. They show that higher levels of cardiorespiratory fitness are generally associated with better quality of life, especially in the domains of physical well-being, school functioning, and peer relationships (16, 17, 56–58). Despite this initial nonlinearity of the relationship with endurance, findings consistently confirm that a child's ability to be physically agile and strong is strongly associated with quality of life and a sense of general well-being (6, 15, 21). The relationship between physical fitness and quality of life is not simple or unambiguous, but may depend on various psychological and social factors, which indicates the complex and potentially contextually conditioned character of this relationship (59).

The specificity of the results in this study may indicate that children of the age studied subjectively perceive demanding endurance tests as excessively strenuous, which may be reflected in their self-assessment of their general condition. Furthermore, it is possible that testing children aged 9–11 years for cardiorespiratory endurance could induce stress and fatigue thereby negatively impacting their subjective quality of life (60). The association between cardiorespiratory fitness (CRF) and health-related quality of life (HRQoL) shows that this relationship is mediated by self-determined motivation (61), meaning that if children lack intrinsic motivation for strenuous tasks, high physical exertion could negatively affect their perceived well-being.

The most important finding of the study comes from the hierarchical regression analysis. Control variables (gender, age, BMI) were not significant predictors of quality of life by themselves, but by introducing physical fitness components, the model becomes statistically significant, confirming that physical fitness components are stronger predictors of quality of life than the biological characteristics included in this study (15, 16, 62). Within these components, cardiorespiratory fitness showed the greatest contribution to explaining the variance in quality of life compared to other physical fitness components and stood out as the most important single predictor in a negative direction (15, 17, 56, 58, 62). Meta-analyses and recent longitudinal studies show that higher cardiorespiratory fitness is associated with better overall HRQoL and especially with physical and psychological well-being, peer relationships and school environment (15, 17, 56, 58). In the study by Morales et al. (63), it was found that cardiorespiratory and muscular fitness were associated with better results in dimensions of physical well-being. In boys, higher levels of cardiorespiratory fitness were associated with better results for physical well-being and social support from peers; In girls, cardiorespiratory fitness was associated with physical well-being, self-perception, social acceptance and the total KIDSCREEN-10 index. Although the KIDSCREEN-10 questionnaire cannot analyze individual dimensions of quality of life, the contribution of cardiorespiratory fitness to the overall level of quality of life can highlight the importance of physical performance that affects well-being (61). These results support the conclusion that while motor speed positively contributes to the psychosocial health of children aged 9–11 by building a sense of competence and energy to cope with everyday challenges in and outside of school, the relationship with cardiorespiratory endurance is more complex and may require a careful balance to avoid physical or psychological overload associated with training and testing for higher endurance (6, 15, 56, 58, 60, 64).

Based on the results obtained, it was confirmed that physical fitness is a significant but limited predictor of quality of life in children aged 9–11 years. Although significant associations were found between individual components of physical fitness and quality of life, their strength is relatively small, which is in line with the conceptualization of quality of life as a complex and multidimensional construct. It is of utmost importance to emphasize that individual components of physical fitness, unlike basic biological and morphological characteristics such as gender, age, and body mass index, showed greater predictive value in explaining quality of life.

At the same time, the observed negative association with quality of life indicates the complexity of this relationship and suggests the need for further research that will include psychological and motivational factors as potential mediators. Overall, the results of this study contribute to the understanding of the relationship between physical fitness and quality of life and confirm that, although physical fitness plays a significant role, it represents only one part of a wider spectrum of factors that shape children's subjective well-being.

Despite the significant results, this study has several limitations that need to be taken into account when interpreting the results. This study was conducted using a cross-sectional design, which does not allow for conclusions about cause-and-effect in the relationship between physical fitness and quality of life; therefore, causal relationships cannot be inferred. Furthermore, the results showed a significant association but not the direction of influence between the analyzed variables. Likewise, quality of life was assessed using a global composite index and a questionnaire (KIDSCREEN-10). It is reliable and practical to use, but it does not provide insight into individual dimensions of quality of life (physical, emotional, social and school), and there is a possibility that specific differences between individual domains of the questionnaire remained hidden in the overall result. Furthermore, the analysis included control variables (gender, age, and BMI) but did not include other potentially important factors that may influence quality of life, such as socioeconomic status, physical activity level, dietary habits, psychological characteristics, or family environment.

Given that quality of life is a complex biopsychosocial construct, the absence of these variables may partially explain the relatively small proportion of explained variance (7.1%), emphasizing that physical fitness is a marginal determinant and the main causes of quality of life variation likely lie in broader psychosocial, socio-economic, and lifestyle factors. Also, the assessment of cardiorespiratory capacity was based on the results of a field test that is often used epidemiologically, but it can be greatly influenced by motivation, especially in children of this age. This may affect the accuracy of the assessment of the actual level of cardiorespiratory fitness. In addition, the sample of respondents was limited to school children aged 9–11 years, which limits the possibility of generalizing the results to other age groups, especially adolescents, where gender differences are more pronounced.

In addition to the limitations, this study has several important advantages. The study was based on a relatively large sample of children of this age, which increases the reliability of the results obtained and allows for more stable statistical estimates. Also, an objective approach to assessing physical fitness was used through standardized field tests, which avoids the limitations of self-assessment and increases the validity of measuring the components of physical fitness. The inclusion of multiple components of physical fitness (cardiorespiratory endurance, speed, explosive and repetitive strength, and agility) provided a comprehensive insight into their relationship with quality of life. Furthermore, the application of hierarchical regression analysis represents a significant methodological advantage because it allows for the assessment of the increase in the value of physical fitness in explaining quality of life, regardless of the influence of control variables (gender, age, and BMI). In this way, it was clearly shown that physical fitness has a greater predictive value compared to gender, age, and body mass index. Future research should use longitudinal designs, include a wider range of psychological and social variables, and analyze individual dimensions of quality of life in order to better understand the mechanisms of the relationship between physical fitness and quality of life in children.

The results obtained have important implications for practice in the field of physical education, education systems, and public health. First of all, the findings indicate the need to systematically encourage the development of physical fitness in children, with special emphasis on a balanced development of cardiorespiratory endurance, speed, and muscle strength, which have been shown to be relevant for quality of life. Physical activity programs in schools should focus not only on developing motor skills, but also on creating a positive experience of movement that avoids excessive physical strain, in order to encourage intrinsic motivation and long-term participation of children in physical activity. The results of this study indicate that the development of physical fitness should be an integral part of strategies and intervention programs aimed at improving the quality of life of children.

## 5 Conclusion

The conducted research provided insight into the complex relationship between physical fitness and quality of life in children aged 9–11 years. The results obtained confirm that physical fitness components explain a significant proportion of the variance in quality of life, while basic demographic and biological parameters do not have this predictive power. In addition, simple movement speed positively predicted HRQoL, whereas cardiorespiratory endurance emerged as a weak negative predictor, suggesting that demanding aerobic tasks may be perceived as a psychological burden at this age. The obtained results emphasize the need to redefine priorities in Physical Education and Health Education teaching in the school system. Emphasizing a balanced, enjoyable approach to movement and skill acquisition over rigorous endurance testing may be a more effective foundation for improving overall psychosocial well-being.

## Data Availability

The raw data supporting the conclusions of this article will be made available by the authors, without undue reservation.
